# Adherence to treatment guidelines and survival in triple-negative breast cancer: a retrospective multi-center cohort study with 9156 patients

**DOI:** 10.1186/1471-2407-13-487

**Published:** 2013-10-21

**Authors:** Lukas Schwentner, Achim Wöckel, Jochem König, Wolfgang Janni, Florian Ebner, Maria Blettner, Rolf Kreienberg, Reyn Van Ewijk

**Affiliations:** 1 Department of Gynecology and Obstetrics, University Ulm, Prittwitzstraße 43, Ulm 89075, Germany; 2 Institute for Medical Biostatistics, Epidemiology and Informatics, University Medical Centre, Johannes Gutenberg University Mainz, Obere Zahlbacher Straße 69, Mainz 55131, Germany

**Keywords:** Breast cancer, Guideline, Survival, Triple negative, Cohort study

## Abstract

**Background:**

Triple-negative breast cancer (TNBC) remains a challenging topic for clinical oncologists. This study sought to evaluate TNBC versus other breast cancer subtypes with respect to survival parameters. We evaluated possible differences in survival in TNBC by age and by the extent to which evidence-based treatment guidelines were adhered.

**Methods:**

This German retrospective multi-center cohort study included 9156 patients with primary breast cancer recruited from 1992 to 2008.

**Results:**

The rates of guideline adherence are significantly lower in TNBC compared to non-TNBC subtypes. These lower rates of guideline adherence can be observed in all age groups and are most pronounced in the >65 subgroup [<50 (20.9% vs. 42.0%), 50–64 (25.1% vs. 51.1%), and >65 (38.4% vs. 74.6%)]. In TNBC patients of all age groups, disease-free survival and overall survival were associated with an improvement by 100% guideline-adherent adjuvant treatment compared to non-adherence. Furthermore, TNBC patients of all ages had similar outcome parameters if 100% guideline-adherent adjuvant treatment was applied.

**Conclusion:**

The rates of guideline-adherent treatment were significantly lower in TNBC, even though guideline adherence was strongly associated with improved survival. In the case of 100% guideline-adherent treatment, no difference in survival was observed over all the age groups examined, even in the group of >65-year-old TNBC patients.

## Background

Breast cancer comprises a complex and heterogeneous group of diseases at the clinical, morphological, and molecular levels [1-6]. Specimens that display basal-like features are called “triple-negative” breast cancer (TNBC) in routine practice because they are defined by their lack of estrogen receptor, progesterone receptors, and human epidermal growth factor receptor 2 (Her2). Although TNBC represents 10-20% of all invasive breast cancers, it is more frequent in young premenopausal and African-American patients [7-9]. TNBC is associated with an advanced stage at initial diagnosis, higher grading, family history, and *BRCA* mutations [7-9]. Additionally, TNBC patients lack the benefit of routinely available targeted therapy, which explains the growing attention of both pathologists and oncologists to an easily recognizable type of breast cancer with aggressive behavior and poor therapeutic options [5].

The prognosis of women with TNBC is significantly poorer compared to that of women with other subtypes of breast cancer, and different routes of metastatic spread may explain the higher recurrence and mortality rates of TNBC patients. The impact of different therapies is yet not clear because few data from randomized controlled trials (RCTs) have been published. Although RCTs are the gold standard of therapy schemes, it is necessary to compare them with observational data.

To date, there is little evidence of the impact of age on survival in the TNBC subgroup. Furthermore, it remains unclear whether internationally validated guidelines are likely to improve survival in TNBC breast cancer patients of different age groups. We, therefore, investigated whether TNBC patients of different age groups benefit from different strategies of therapy (e.g., surgery, radiation, adjuvant chemotherapy) according to international evidence-based guidelines. We analyzed the association between guideline-adherent adjuvant treatment and survival outcome in TNBC by investigating the impact of different guideline-adherent therapies on the survival (OAS and DFS) of TNBC patients in an observational, retrospective, multicenter-study called “Breast Cancer Care under Evidence-based Guidelines” (BRENDA).

## Methods

In this retrospective, multi-center cohort study, we analyzed data from 9156 patients with primary breast cancer diagnosed or treated at the Department of Gynaecology and Obstetrics at the University of Ulm and 16 partner clinics (all certified by the German Society of Cancer as breast cancer centers) in Baden-Württemberg (Germany) between 1992 and 2008. A new documentation system called BRENDA was designed and used for this purpose. This documentation system included a retrospective chart review to abstract TNM stage, histological subtype, grading, lymphatic and vascular invasion, estrogen, progesterone, and Her2 expression, date of diagnosis, and all adjuvant therapies. Data on adjuvant therapies, including surgery (date of surgery, BCT, mastectomy, sentinel-node biopsy, and axillary lymph node dissection), adjuvant systemic chemotherapy, adjuvant endocrine therapy, and detailed information on administered adjuvant radiotherapy were also collected. In the follow-up, data on first recurrences, secondary primary tumors, and date and cause of death were obtained. Questionnaires were sent to the physicians involved in the follow-up care, to local death registrars, and to patients to determine the recurrence and survival status of the patients. As measures of comorbidity, the American Society of Anesthesiologists (ASA) Physical Status and the New York Heart Association (NYHA) cardiac score at the time of surgery were collected for the patients, if available. Furthermore, occurrences of myocardial infarction, stroke, and malignant diseases were recorded.

Written informed consent was obtained from all patients included in this clinical study. The inclusion criterion was histologically confirmed invasive breast cancer. The exclusion criteria were carcinoma in situ, primary metastatic disease, bilateral breast cancer, primary occult disease, phylloides tumor, incomplete follow-up, unknown Her2 status or hormone receptor status (HR status), or missing data on variables used as covariates in the survival analyses. We considered triple-negative breast cancer as being estrogen receptor- and progesterone receptor-negative (IRS 0) and Her2 negative (0, 1+, 2+, and FISH-negative) [10].

The definition of evidence-based guideline-adherent adjuvant treatment was based on internationally evidence based validated guidelines. Wolters et al. demonstrated that the treatment recommendations within international guidelines are identical and differ only marginally in adjuvant endocrine therapy [11]. We, therefore, decided to base the definition of guideline-adherent adjuvant treatment on the German national consensus guideline (S3 guideline) for the decision of loco-regional treatment (surgery and radiotherapy), chemotherapy, and endocrine therapy (only non-TNBC) [12] unless it was one of the guidelines taken into consideration by Wolters et al. [11]. All of the applied therapy regimens were retrospectively evaluated concerning their adherence to the S3 guideline. We classified the adjuvant treatments as surgery, chemotherapy, and radiotherapy. Omission of any of the suggested adjuvant treatment or abandonment of any adjuvant treatment was classified as non-compliance with the suggested adjuvant therapy, resulting in one ore more guideline violations (GV) for each patient (see Table 1).

**Table 1:** Inclusion criteria for guideline adherence based on the German national consensus guideline (S3 guideline) for the decisions regarding loco-regional treatment (surgery and radiotherapy), chemotherapy, and endocrine therapy

**Group A – Surgical therapy**		
	**Breast conserving therapy** (Reference: Statements 7, 8)	
	**Conforming to guideline recommendations**	**Non-conforming to guideline recommendations**
	BCT in DCIS and LCIS < 4 cm	BCT when tumor size > 4 cm
	BCT in R0	BCT in R1
		BCT in presence of multicentricity
		BCT in presence of inflammatory carcinoma
	**Mastectomy** (Reference: Statement 9)	
	**Conforming to guideline recommendations**	**Non-conforming to guideline recommendations**
	Mastectomy for microcalcification of malignant type	No mastectomy in the presence of microcalcification of malignant type
	Mastectomy for intraductal carcinoma and tumor size > 4 cm	No mastectomy in the presence of multicentricity
	Mastectomy for multicentricity	Mastectomy for intraductal carcinoma with a tumor size < 4 cm
	Mastectomy for R1	No mastectomy for inflammatory breast cancer
	Mastectomy for inflammatory breast cancer	
	**Axillary dissection** (Reference: Statements 12, 13)	
	**Conforming to guideline recommendations**	**Non-conforming to guideline recommendations**
	Removal of invasive carcinoma + dissection for at least level I and II + removal of at least 10 lymph nodes	Lymph node removal in non-invasive carcinoma
		Invasive carcinoma + (only dissection for level I or removal of <10 lymph nodes)
**Group B – Radiotherapy**		
	**Radiotherapy secondary to BCT** (Reference: Statements 23, 24)	
	**Conforming to guideline recommendations**	**Non-conforming to guideline recommendations**
	Radiotherapy secondary to BCT for invasive carcinoma	No radiotherapy secondary to BCT for invasive carcinoma
	**Postmastectomy strategy** (Reference: Statements 25, 26)	
	**Conforming to guideline recommendations**	**Non-conforming to guideline recommendations**
	Radiotherapy secondary to mastectomy and R1/R2	Radiotherapy in mastectomy and R0
	Radiotherapy secondary to mastectomy and nodes involved ≥4	Radiotherapy in mastectomy and T = T1 or T2
	Radiotherapy T = T3 or T4	No radiotherapy in mastectomy and R1/R2
		No radiotherapy in mastectomy and nodes involved ≥4
		No radiotherapy in T = T3 or T4
**Group C –Endocrine therapy**		
	**Endocrine therapy** (Reference: Statements 33–37)	
	**Conforming to guideline recommendations**	**Non-conforming to guideline recommendations**
	Tamoxifen for invasive carcinoma in patients with positive hormone receptor status	Hormone therapy in receptor-negative patients
	GnRH + tamoxifen or GnRH in premenopausal patients with positive hormone receptor status	
	Postmenopausal patient and positive hormone receptor status and tamoxifen or aromatase inhibitor	
	Endocrine therapy after chemotherapy in positive receptor status	
	Tamoxifen for DCIS	
*When estrogen receptor (ER) and progesterone receptor (PgR) are negative:*		
Risk group	Chemotherapy	Guideline conformity
**Low**	CT performed	Overtherapy
	No CT performed	Guideline conformity
**Moderate**	CMF/EC/AC	Undertherapy
	FEC/T	Guideline conformity
	No CT performed	Undertherapy
**High**	CMF/EC/AC	Undertherapy
	FEC/T	Guideline conformity
	No CT performed	Undertherapy
*ER and PgR >0 and <6*		
Risk group	Chemotherapy	Guideline conformity
**Low**	CT performed	Overtherapy
	No CT performed	Guideline conformity
**Moderate**		
**Premenopausal**	CT performed	Guideline conformity
	No CT performed	Guideline conformity
**Postmenopausal**	CT performed	Guideline conformity
	No CT performed	Undertherapy
**High**	CMF/EC/AC	Undertherapy
	FEC/T	Guideline conformity
	No CT performed	Undertherapy
*ER or PgR ≥6*	Chemotherapy	Guideline conformity
Risk group		
**Low**	CT performed	Overtherapy
	No CT performed	Guideline conformity
**Moderate**	CT performed	Guideline conformity
	No CT performed	Guideline conformity
**High**	CMF/EC/AC	Undertherapy
	FEC/T	Guideline conformity
	No CT performed	Undertherapy

Risk group classification in the 2008 S3 guideline is according to Goldhirsch et al. [27].

Key: *CT*, Chemotherapy; *CMF*, Cyclophosphamide, methotrexate, fluorouracil, *EC/AC*, Epirubicin, cyclophosphamide/adriamycin, cyclophosphamide, *FEC,* Fluorouracil, epirubicin, cyclophosphamide, *T*, Taxane.

### Statistical analysis

Descriptive analyses were first performed. The data are presented separately for the group of TNBC and the group of non-TNBC, and the *P*-values for the comparisons between both groups are based on χ^2^-tests or t-tests; a *P*-value of 0.05 (two-tailed test) was considered significant. To estimate hazard ratios (HRs) and confidence intervals (CIs), multivariate survival regressions were then performed using frailty models with a gamma-distributed random term [13]. The primary endpoints were disease-free survival (DFS) and overall survival (OAS). All of the regression models were adjusted for tumor size (4 categories), grading (3 categories), nodal status (0, 1–3, or 4+ positive nodes), menopausal status (binary), year of diagnosis, comorbidities (see below), presence or absence of information on comorbidities, and age (with the exception of the analyses that were stratified by age group). Comorbidities were measured using the scale of the ASA (ASA 3 or greater), the scale of the NYHA (NYHA class 3 or higher), history of any prior cancers, and history of apoplexy, transient ischemic attack (TIA), or myocardial infarction. Survival function plots were derived from the survival regression analysis.

### Ethical approval

This study and the BRENDA project have been approved by the Ethics Committee of the University of Ulm, which coveres all participating breast cancer centers of the BRENDA network.

## Results

**Figure 1:**
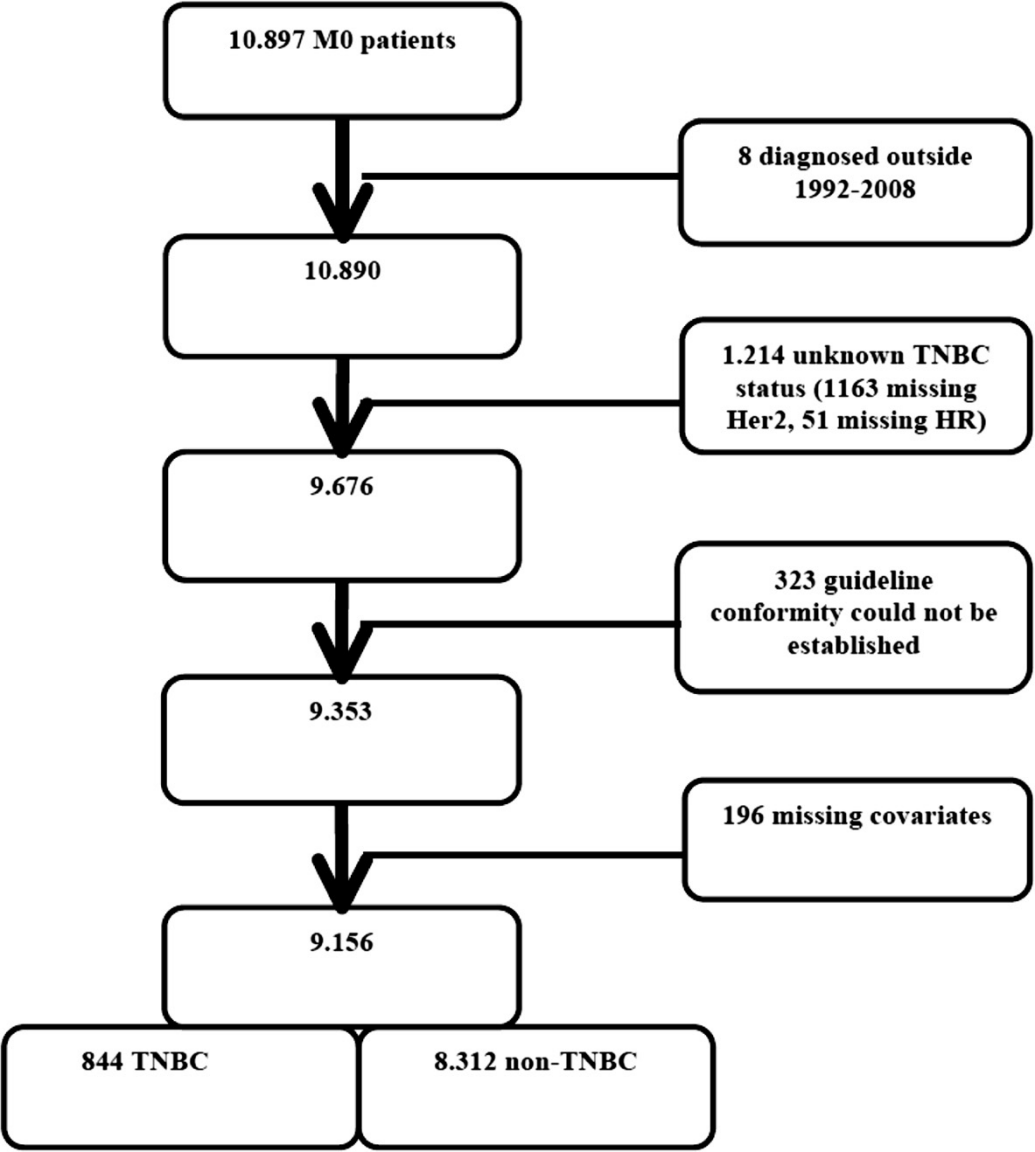
Cohort diagram of the study group.

The investigated cohort consisted of 10,897 breast cancer patients. In total, 1741 patients were excluded after applying the exclusion criteria: 8 patients had initially been diagnosed outside of the 1992–2008 time frame or had an unknown date of death; the TNBC status of 1214 patients was unknown (1163 unknown Her2 status, 51 unknown HR status); guideline conformity could not be established for 323 patients; and data for the covariates used in our analyses were missing for the remaining 196 patients. The final cohort consisted of 9156 patients with histologically confirmed invasive breast cancer (see Figure 1). In the final cohort, 844 patients (9.2%) had TNBC [median age: 57.7 (range: 27–97)].

Because we employed a multi-center design, we also compared the different participating centers. In the cohort of the university department, the patients were significantly younger (59.8 vs. 61.6 years), and G3 was significantly more common (31.2% vs. 27.3%). However, HR-positive breast cancers occurred significantly more often in the university department, and the patients in the university department showed significantly higher rates of comorbidities as measured by the ASA and NYHA score. In the case of TNBC prevalence, we could not demonstrate a significant difference between the university department and the participating breast cancer centers.

Compared to the remaining individuals (women without TNBC) (8312 breast cancer patients; 90.8%), the TNBC subgroup showed a significantly higher grade (*P* < 0.001), was more frequently premenopausal (*P* < 0.001), and more frequently received adjuvant chemotherapy (43.0% vs. 77.1%; *P* < 0.001). The baseline characteristics of the 9156 patients are shown in Table 2. Surprisingly, 22.9% of the TNBC subgroup did not receive any adjuvant chemotherapy.

**Table 2:** Baseline characteristics of the study group based on 8312 non-TNBC patients and 844 TNBC patients included in the study

	**Non-TNBC**		**TNBC**			
	**8312 (90.8%)**		**844 (9.2%)**			
	***Mean***	***N***	***Mean***	***N***	***p-value***	
*Age (range)*	61.2 (22; 97)	8312	57.7 (27; 97)	844	<0.001	
*Postmenopausal*	74.8%	6220	66%	557	<0.001	
*T 1*	55.3%	4600	48.5%	409	<0.001	
*T 2*	36%	2995	41.4%	349		
*T 3*	3.8%	313	5.9%	50		
*T4*	4.9%	404	4.3%	36		
*Node negative*	59.9%	4979	62.6%	528	0.133	
*1-3*	23.8%	1978	20%	169		
*>3*	16.3%	1355	17.4%	147		
*G 1*	9.9%	820	1.4%	12	<0.001	
*G 2*	65.5%	5447	25.4%	214		
*G 3*	24.6%	2045	73.2%	618		
*ASA Score >3*	21.7%	1068	18.3%	94	0.072	
*NYHA Class > III*	3.5%	92	4.0%	12	0.652	
*Myocardial infarction, stroke, or TIA*	4.2%	230	3.4%	19	0.340	
*HR negative*	5.8%	481	100.0%	844	<0.001	
*HR IRS 1-5*	23.5%	1953	0.0%	0		
*HR positive*	70.7%	5878	0.0%	0		
*Her2neu positive*	17.7%	1468	0.0%	0	<0.001	
*Endocrine therapy*	82.0%	6816	5.6%	47	<0.001	
*Chemotherapy*	43.0%	3575	77.1%	651	<0.001	
*Mastectomy*	29.0%	2414	26.7%	225	0.145	
*Radiotherapy*	79.2%	6579	80.3%	678	0.420	

ASA, NYHA, and myocardial infarction/stroke/TIA were available for 4911, 2608, and 5466 non-TNBC patients and for 513, 297, and 564 TNBC-patients, respectively.

**Figure 2:**
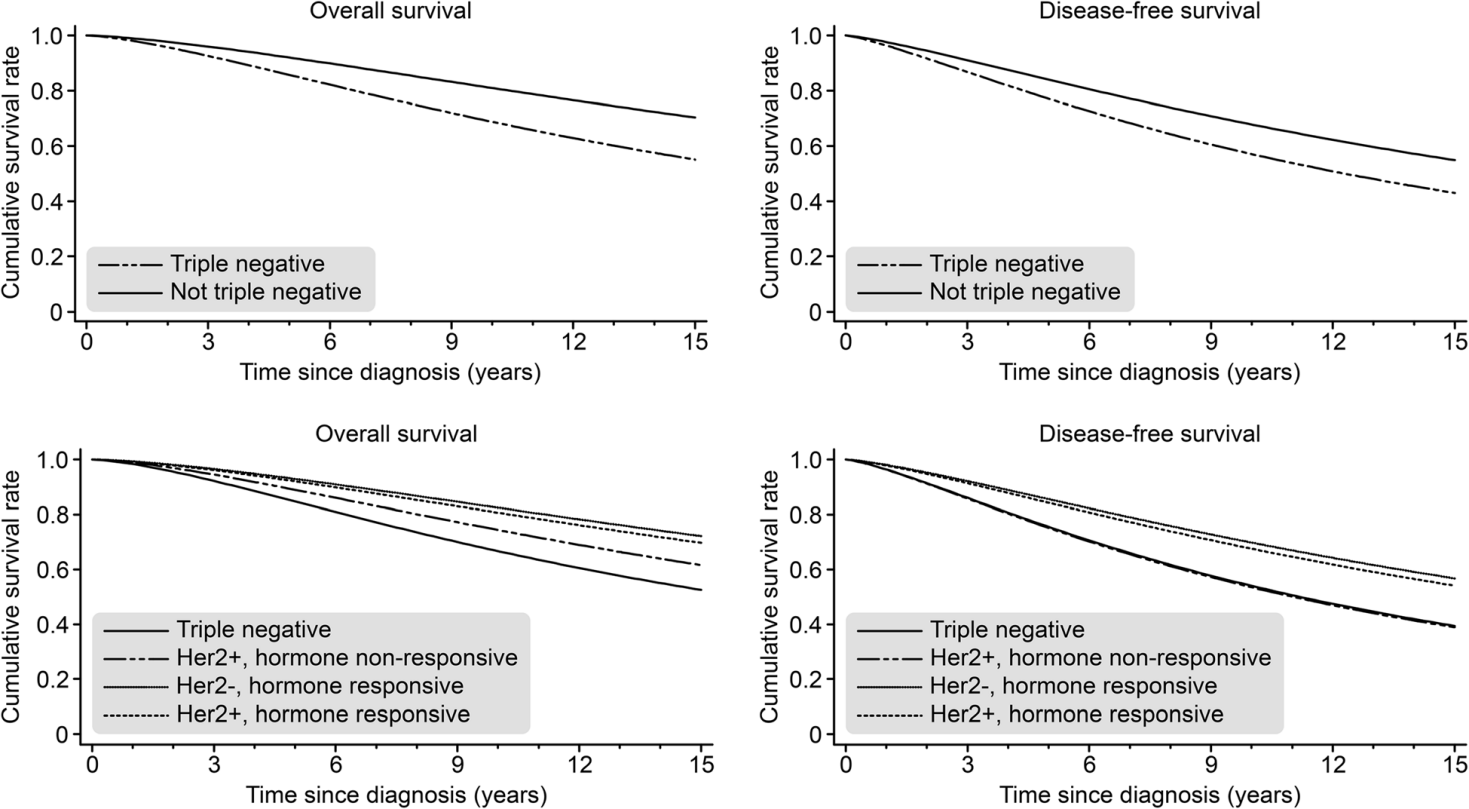
Overall survival (OAS) and disease-free survival (DFS) for TNBC (n = 844) versus non-TNBC (n = 8.312) and for TNBC versus the groups defined by HR status and Her2 status (adjusted for year of diagnosis, tumor size, grading, nodal status, menopausal status, and comorbidity).

Initially, we attempted to identify the impact of TNBC on survival parameters and, therefore, compared the TNBC subgroup with non-TNBC patients. The TNBC subgroup demonstrated significantly decreased OAS [HR = 1.92; *P* < 0.001] and DFS [HR = 1.53; *P* < 0.001] values compared to the non-TNBC population (see Figure 2). Because breast cancer is a complex disease at the clinical and morphological levels, we also investigated the differences between different breast cancer phenotypes (HR+/Her2-, HR+/Her2+, HR-/Her2+). The TNBC subgroup showed a significant decrease in OAS and DFS compared to the HR+/Her2- [OAS, HR = 0.42; *P* < 0.001; DFS, HR = 0.53; *P* < 0.001] and HR+/Her2+ breast cancer subtypes [OAS, HR = 0.48; *P* < 0.001; DFS, HR = 0.59; *P* < 0.001]. Compared to HR-/Her2+ breast cancer, only OAS [HR = 0.69; *P* = 0.015] showed a significant impairment in TNBC, whereas DFS [HR = 1.02; *P* = 0.88] was not significantly different (see Figure 2).

**Figure 3:**
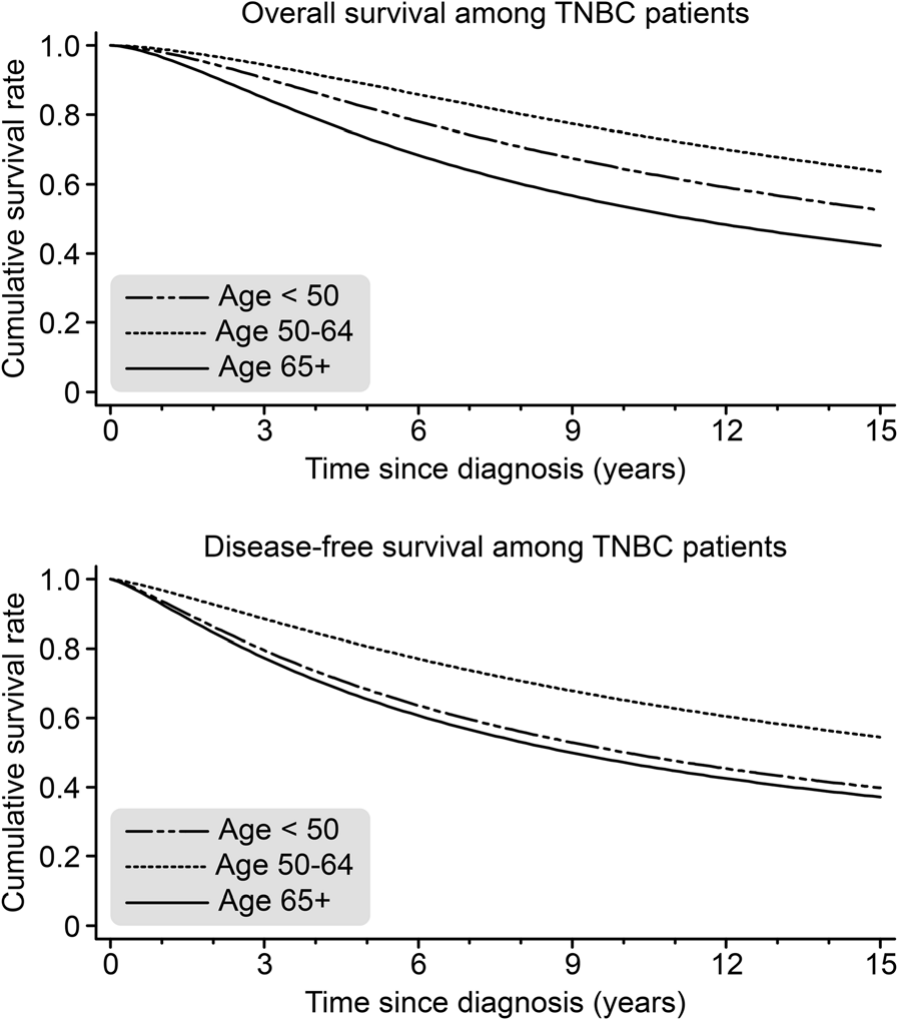
Comparison of overall survival (OAS) and disease-free survival (DFS) among TNBC patients in three age groups (<50 n = 283; 50–64 n = 274; >65 N = 287) (adjusted for year of diagnosis, tumor size, grading, nodal status, menopausal status, and comorbidity).

Furthermore, we investigated the TNBC patients in different age groups (<50, 50–64, and ≥65) (see Table 3). The TNBC patients aged ≥65 showed significantly higher rates of comorbidities, as measured by ASA (*P*-value for the comparison of the three age groups: *P* < 0.001) and NYHA (*P* = 0.030) and of myocardial infarction/stroke/TIA (*P* = 0.001). These patients also received chemotherapy [90.8% (<50), 86.5% (50–64), and 54.7% (≥65)] (*P* < 0.001) and radiotherapy [86.2%(<50), 88.0% (50–64), and 67.2% (≥65)] (*P* < 0.001) less often; in contrast, they underwent mastectomy significantly more often [18.7% (<50), 21.5% (50–64), and 39.4% (≥65)] (*P* < 0.001) and showed significantly more locally advanced (T4) tumors (*P* < 0.001) (see Table 2). When comparing the three age groups, we observed that the patients aged ≥65 had a significantly worse OAS [HR = 0.31; *P* < 0.001] and DFS [HR = 0.42; *P* < 0.001] compared to the TNBC patients aged 50–64 (see Figure 3). However, the difference between TNBC ≥65 and TNBC <50 was not significant for OAS [HR = 0.56; *P* = 0.231] or DFS [HR = 0.87; *P* = 0.732].

**Table 3:** Baseline characteristics of the TNBC age subgroups (<50, 50–64, and ≥65)

**TNBC**	**<50 (283)**	**50-64 (274)**	**≥65 (287)**	
	***Mean***	***Mean***	***Mean***	***p-value***
*Age (mean, range)*	41.9 (27; 50)	57.7 (50; 65)	73.3 (65; 97)	<0.001
*Postmenopausal*	9.9%	88.3%	100.0%	<0.001
*T 1*	58%	47.8%	39.7%	<0.001
*T 2*	34.6%	42%	47.4%	
*T 3*	6.4%	6.2%	5.2%	
*T 4*	1.1%	4%	7.7%	
*Node negative*	66.8%	61.7%	59.2%	0.165
*1-3*	19.8%	21.2%	19.2%	
*>3*	13.4%	17.2%	21.6%	
*G 1*	0.7%	2.2%	1.4%	0.012
*G 2*	18.7%	29.2%	28.2%	
*G 3*	80.6%	68.6%	70.4%	
*ASA Score* ≥*3*	5.5%	13.1%	37.8%	<0.001
*NYHA Class* ≥ *III*	0.9%	4.1%	8.3%	0.030
*Myocardial infarction, stroke, or TIA*	0.5%	2.7%	7.1%	<0.001
*Endocrine therapy*	5.7%	6.6%	4.5%	0.573
*Chemotherapy*	90.8%	86.5%	54.7%	<0.001
*Mastectomy*	18.7%	21.5%	39.4%	<0.001
*Radiotherapy*	86.2%	88.0%	67.2%	<0.001

The *p*-values are based on differences between the reference groups using the χ^2^-test and (for age) the t-test.

### Guideline adherence in the TNBC group and TNBC age subgroups

Guideline adherence was significantly lower in the TNBC patients compared to the non-TNBC population (*P* < 0.001) (see Table 4). Similarly, the TNBC patients showed a significantly lower guideline adherence (*P* < 0.001) in all the age groups, even the TNBC patients <50, who had lower rates of comorbidities than the other two subgroups (*P* < 0.001). These differences in guideline adherence were mainly caused by significantly higher rates of guideline violations concerning chemotherapy in all the TNBC age groups (*P* < 0.001) (see Table 3). This deficiency in guideline adherence concerning adjuvant chemotherapy was most pronounced in the ≥65 TNBC subgroup (69.3% versus 23.0% chemotherapy guideline violations) (*P* < 0.001). All of the other investigated adjuvant treatment modalities (surgery and radiotherapy) did not show significant differences between the TNBC and non-TNBC populations (*P* > 0.05).

**Table 4:** Number and percentage of guideline violations (GV) among non-TNBC and TNBC age subgroups (complete sample, <50, 50–64, and ≥65) stratified for all adjuvant treatment modalities (surgery, chemotherapy, and radiotherapy)

**All patients**	**Non-TNBC**		**TNBC**		
	**N = 8,312 (90.8%)**		**N = 844 (9.2%)**		
	**%**	***N***	**%**	***N***	***p-value***
*≥1 GV*	29.8%	2476/8312	56.0%	473/844	<0.001
*GV radiotherapy*	8.1%	671/8312	10.0%	84/844	0.059
*GV surgery*	13.2%	1095/8312	13.5%	114/844	0.785
*GV chemotherapy*	13.4%	1117/8312	46.6%	393/844	<0.001
***Patients < 50***	**Non-TNBC**		**TNBC**		
	**N = 1,784 (86.3%)**		**N = 283 (13.7%)**		
	**%**	***N***	**%**	***N***	***p-value***
*≥1 GV*	20.9%	372/1784	42.0%	119/283	<0.001
*GV radiotherapy*	5.0%	90/1784	7.1%	20/283	0.159
*GV surgery*	15.8%	281/1784	13.8%	39/283	0.395
*GV chemotherapy*	1.9%	34/1784	30.0%	85/283	<0.001
***Patients 50-64***	**Non-TNBC**		**TNBC**		
	**N = 3,046 (91.7%)**		**N = 274 (8.3%)**		
	**%**	***N***	**%**	***N***	***p-value***
*≥1 GV*	25.1%	766/3046	51.1%	140/274	<0.001
*GV radiotherapy*	5.0%	153/3046	6.6%	18/274	0.267
*GV surgery*	13.8%	420/3046	14.6%	40/274	0.710
*GV chemotherapy*	9.2%	281/3046	39.8%	109/274	<0.001
***Patients ≥65***	**Non-TNBC**		**TNBC**		
	**N = 3,482 (92.4%)**		**N = 287 (7.6%)**		
	**%**	***N***	**%**	***N***	***p-value***
*≥1 GV*	38.4%	1338/3482	74.6%	214/287	<0.001
*GV radiotherapy*	12.3%	428/3482	16.0%	46/287	0.067
*GV surgery*	11.3%	394/3482	12.2%	35/287	0.652
*GV chemotherapy*	23.0%	802/3482	69.3%	199/287	<0.001

The *p*-values are derived from χ^2^-tests.

**Figure 4:**
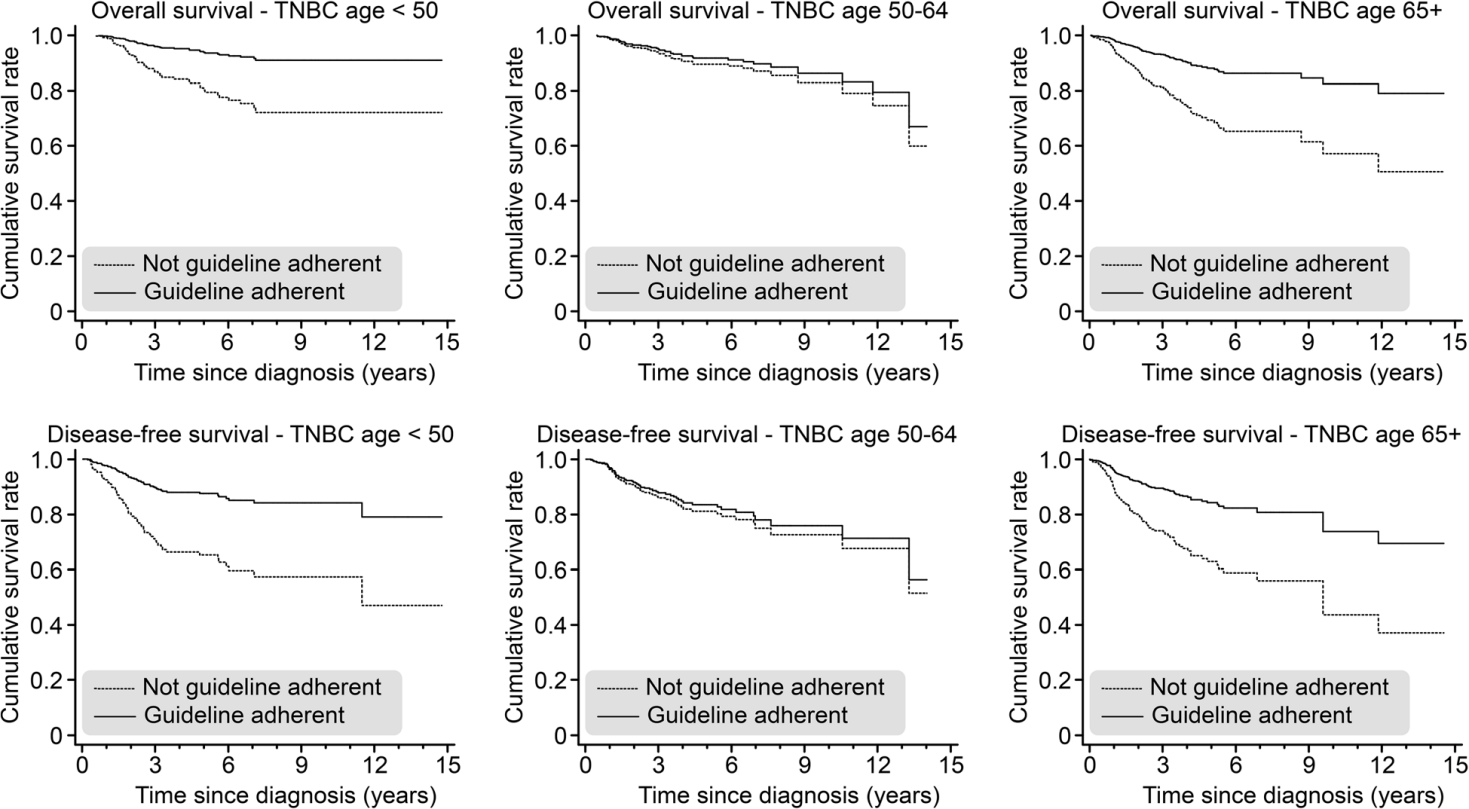
Overall survival (OAS) and disease-free survival (DFS) for TNBC patients who received (versus those who did not receive) 100% guideline-adherent adjuvant treatment, as stratified by age (<50, 50–64, and ≥65) and adjusted for year of diagnosis, tumor size, grading, nodal status, menopausal status, and comorbidities.

Next we compared the survival of TNBC patients in the three age groups according to guideline adherence (one or more guideline violations vs. complete guideline adherence). In all three age groups, patients treated according to guidelines had a better OAS and a better DFS (see Figure 4), and this effect was significant for the youngest and oldest age groups: OAS in the ≥65 TNBC subgroup: HR = 2.89 (*P* = 0.001); DFS: HR = 2.72 (*P* = 0.001); OAS in the <50 TNBC subgroup: HR = 3.47 (*P* = 0.001); DFS: HR = 3.20 (*P* < 0.001); OAS in the 50–64 TNBC subgroup: HR = 1.27 (*P* = 0.515); DFS: HR = 1.16 (*P* = 0.633).

**Figure 5:**
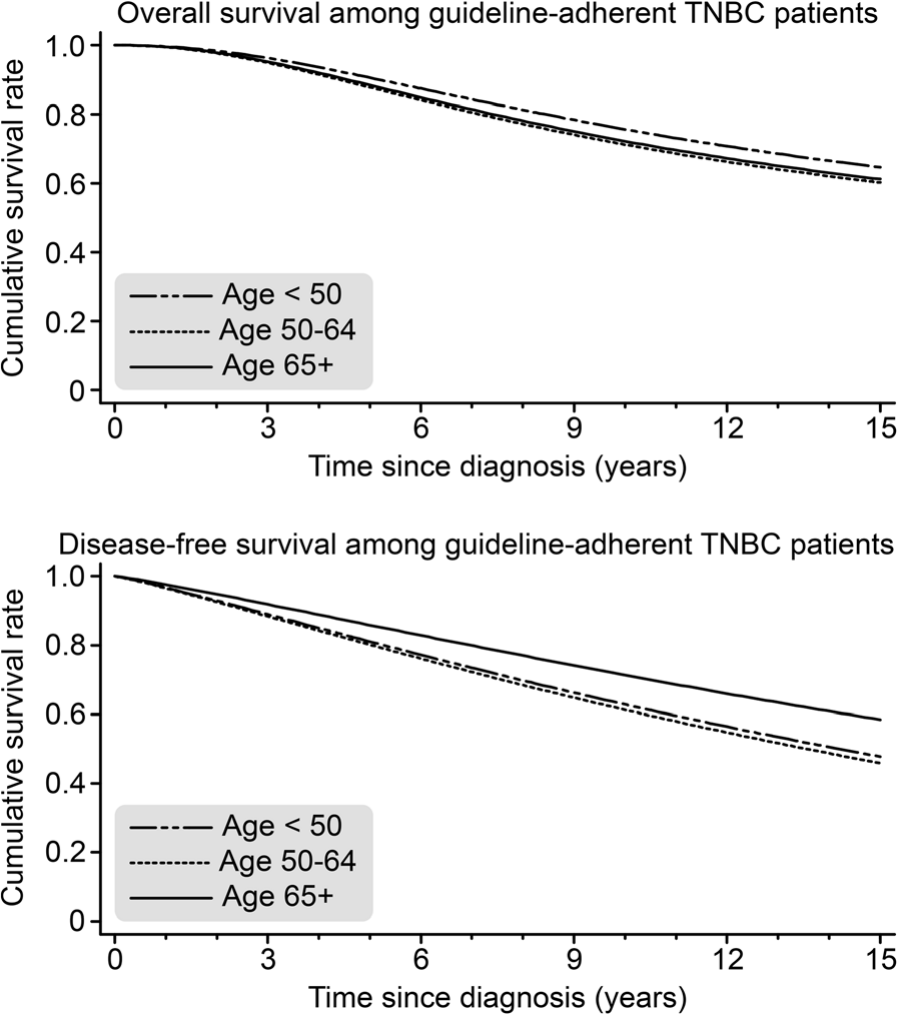
Overall survival (OAS) and disease-free survival (DFS) for TNBC patients who received 100% guideline-adherent adjuvant treatment, as stratified by age (<50 n = 164; 50–64 n = 134; ≥65 n = 73) and adjusted for year of diagnosis, tumor size, grading, nodal status, menopausal status, and comorbidities.

We also investigated the differences between the 100% guideline-treated TNBC patients in those age groups (<50, 50–64, and ≥65). We chose the TNBC ≥65 group as a reference group and compared it to both the TNBC 50–64 [OAS, HR = 0.76; *P* = 0.803; DFS, HR = 1.38; *P* = 0.602] and <50 [OAS, HR = 1.08; *P* = 0.909; DFS, HR = 1.45; *P* = 0.331] subgroups. We did not observe a significant difference in any outcome parameter by age group under the condition of 100% guideline-adherent treatment (see Figure 5). However, if we compared all the 100% guideline-adherent TNBC patients with all the 100% guideline-adherent non-TNBC patients, we observed a significantly inferior OAS [HR = 01.84; *P* = 0.004] and DFS [HR = 1.55; *P* = 0.012] in the patients suffering from TNBC.

## Discussion

TNBC remains a challenge in breast cancer care for clinical oncologists. Although evidence-based guidelines are known to be beneficial in the TNBC subtype [14], to our knowledge, very few investigations have studied the influence of age on the prognosis of TNBC [15]. Furthermore, the efficacy of breast cancer guidelines in elderly TNBC patients has explicitly been investigated to date and has only been investigated in non-TNBC patients [16,17].

In the present study, we observed significantly inferior survival parameters in the TNBC cohort and in the different age groups of this cohort. The TNBC ≥65 subgroup demonstrated the worst survival. Guideline violations occurred significantly more often in the TNBC subgroups, particularly in the TNBC ≥65 subgroup. However, when comparing only those TNBC patients who were treated according to the guidelines, we found no significant differences in OAS or DFS between the age groups, indicating that guideline adherence is associated with improved survival parameters in primary TNBC patients overall different age groups. Conversely, the patients who did not undergo strict guideline-adherent treatment had a significant impairment in survival parameters, except for the 50–64 TNBC age group. Significantly higher rates of guideline violations concerning adjuvant chemotherapy could be observed in TNBC and it is, therefore, likely that these are the most important reasons for the inferior survival. Hence, it is most likely that the higher rates of chemotherapy guideline violations in the ≥65 TNBC patients contributed to their inferior survival [14,15].

There are several other factors, such as higher rates of comorbidities, that could explain the inferior survival in the ≥65 age group. Indeed, there is a clear association between comorbidities and guideline adherence. Our study is an observational study, and confounding factors may have influenced both the treatment options (or guideline conformity) and survival. We observed a high percentage of non-guideline-adherent adjuvant treatment (51.1%) in the 50–64 TNBC subgroup, which was between the percentages of guideline-non-adherent therapy for the other age groups (42.0% for the <50 and 74.6% for ≥65 subgroups). In addition to the higher rates of comorbidities, the factors that might explain this result are the relatively low rates of G3 tumors (68.6%) in the 50–64 TNBC subgroup, the fact that 86.5% of the 50–64 TNBC subgroup received some type of chemotherapy (not all guideline-adherent, see Table 2), and the fact that 61.7% of the 50–64 TNBC subgroup were node negative. In summary, the 50–64 TNBC subgroup demonstrated a relatively low risk profile (a low rate of G3 and a high rate of node-negative patients) compared to the other TNBC subgroups, and 86.5% of these patients received any type of adjuvant chemotherapy; however, those chemotherapies were not all guideline-conforming therapies. Clearly, these circumstances lower the effects of guideline-adherent adjuvant treatment, leading to non-significant improvement in survival parameters.

Only a few clinical research studies have investigated the impact of guideline-adherent therapeutic regimens on clinical outcomes [17-19]. These studies have confirmed that there appears to be a strong association between guideline-adherent treatment and improved survival, particularly for several subtypes of breast cancer [20-23]. Although both patient-related and physician-related factors can preclude guideline-adherent treatment, comorbidities are probably among the most important of these factors [24,25]. Indeed, comorbidities are one of the most important reasons why elderly breast cancer patients are not able to follow strict guideline-adherent therapy pathways, which is one of the reasons explaining their unfavorable outcome. Unfortunately, the present study cannot completely corroborate the significance of comorbidities for guideline adherence, as only NYHA and ASA scores were recorded. It is therefore necessary to address this issue in a prospective trial, which reduces confounding factors and allows measurement of comorbidities in a validated scoring system. Another point to consider is that some patients are not willing to follow strict adjuvant treatment pathways, and this factor was not recorded in this study either. In this specific cohort, all of the patients are treated in specialized and certified interdisciplinary breast cancer centers for which an interdisciplinary tumor board, for example, is a requirement for certification by the German Cancer Society. Several other factors influence guideline adherence in breast cancer, such as education, access to medical resources, health care services themselves, and an urban vs. rural location [26]. Hence, in the current retrospective study, there are confounding factors affecting both the treatment and outcome parameters. To reduce the effects of these potentially confounding factors, we controlled our analyses for the most important prognostic factors (i.e., age, affected lymph nodes, grading, hormone receptor status, menopause status, year of diagnosis, treatment in a university hospital, tumor size, and comorbidity) and included a shared frailty term in our survival regressions.

An important methodological difficulty of the present study is the retrospective nature of data collection. It is, therefore, only possible to draw associations between guideline-adherent treatment and favorable outcome parameters. Drawing valid causal conclusions concerning survival parameters would only be appropriate if treatment allocations were randomized and prospective. However, randomization concerning guideline-adherent treatment is not viable because we cannot assign guideline-adherent and non-guideline-adherent therapeutic regimens at random to patients.

## Conclusion

In summary, our data suggest that TNBC has an important impact on survival among breast cancer patients and remains the most challenging subtype of invasive breast cancer. Although its incidence in very young breast cancer patients is relatively high [7], an age ≥65 is associated with an unfavorable outcome in TNBC in this analysis. However, guideline-adherent adjuvant treatment is associated with a significant improvement in survival parameters in TNBC patients <50 and ≥65 years old. All the TNBC age groups demonstrated an equally favorable outcome if guideline-adherent treatment was applied, even those aged ≥65. Although guideline-adherent adjuvant treatment significantly improves survival in TNBC, the survival parameters in guideline-adherent non-TNBC patients remain significantly better. Accordingly, there is an urgent need to improve therapeutic strategies toward following internationally validated evidence-based guidelines. It is unknown why so many patients cannot follow a guideline-adherent adjuvant treatment pathway. Future research to determine why many patients fail to adhere to therapeutic guidelines may represent a profitable research area that could result in improved survival.

## Competing interest

All the authors declare that there are no potential conflicts of interest, including any financial, personal, or other relationship with other people or organizations that could inappropriately influence this work.

## Authors’ contributions

LS Idea, data collection, draft, study design. AW Idea, data collection, draft, study design. JK statistical analysis. WJ Data collection, draft, study design. FE Draft. MB Idea, data collection, draft, study design, statistical analysis. RK Idea, data collection, draft, study design, statistical analysis. RVE Idea, data collection, draft, statistical analysis. All authors read and approved the final manuscript.

## Pre-publication history

The pre-publication history for this paper can be accessed here:

http://www.biomedcentral.com/1471-2407/13/487/prepub

## References

[B1] Badve S Dabbs DJ Schnitt SJ Baehner SL Decker T Eusebi V Fox SB Ichihara S Jacquemier J Lakhani SR Palacios J Rakha EA Richardson AL Schmitt FC Tan PH Tse GM Weigelt B Ellis IO Reis-Filho JS Basal-like and triple-negative breast cancers: a critical review with an emphasis on the implications for pathologists and oncologists Mod Pathol 2011 24 2 157 167 10.1038/modpathol.2010.200 21076464

[B2] Rakha EA Ellis IO Triple-negative/basal-like breast cancer: review Pathology 2009 41 1 40 47 10.1080/00313020802563510 19089739

[B3] Rakha EA El-Sayed ME Green AR Lee AH Robertson JF Ellis IO Prognostic markers in triple-negative breast cancer Cancer 2007 109 1 25 32 10.1002/cncr.22381 17146782

[B4] Hudis CA Gianni L Triple-negative breast cancer: an unmet medical need Oncologist 2011 16 1 1 11 10.1634/theoncologist.2010-0301 21278435

[B5] Teng YH Thike AA Wong NS Tan PH Therapeutic targets in triple negative breast cancer-where are we now? Recent Pat Anticancer Drug Discov 2011 6 2 196 209 10.2174/157489211795328521 21247402

[B6] Pal SK Childs BH Pegram M Triple negative breast cancer: unmet medical needs Breast Cancer Res Treat 2011 125 3 627 636 10.1007/s10549-010-1293-1 21161370 PMC3244802

[B7] Loibl S Jakisch C Gade S Untch M Paepke S Kuemmel S Schneeweiss A Jakisch C Huober J Hilfrich J Hanusch C Gerber B Eidtmann H Denkert C Costa SD Blohmer JU Nekljudova K Mehta K Von Minckwitz G Neoadjuvant chemotherapy in the very young breast cancer patients Cancer Res 2012 72 Suppl. 24 S3-1 (SABCS 2012)

[B8] Boyle P Triple-negative breast cancer: epidemiological considerations and recommendations Ann Oncol 2012 23 Suppl. 6 vi7 vi12 23012306 10.1093/annonc/mds187

[B9] Lee E McKean-Cowdin R Ma H Spicer DV Van Den Berg D Bernstein L Ursin G Characteristics of triple-negative breast cancer in patients with a BRCA 1 mutation: results from a population-based study of young women J Clin Oncol 2011 29 33 4373 4380 10.1200/JCO.2010.33.6446 22010008 PMC3221522

[B10] Remmele W Stegner HE Recommendation for uniform definition of an immunoreactive score (IRS) for immunohistochemical estrogen receptor detection (ER-ICA) in breast cancer tissue Pathologe 1987 8 3 138 140 3303008

[B11] Wolters R Regierer AC Schwentner L Geyer V Possinger K Kreienberg R Wischnewsky MB Wöckel A A comparison of international breast cancer guidelines—do the national guidelines differ in treatment recommendations? Eur J Cancer 2012 48 1 1 11 10.1016/j.ejca.2011.06.020 21741830

[B12] Kreienberg R Kopp I Albert U Bartsch HH Beckmann MW Berg D Bick A du Bois A Budach A Dunst J Engel J Ernst B Geraedts M Henscher U Hölzel D Jackisch C König K Kreipe H Kühn T Lebeau A Leinung S Link H Lück HJ Madjar H Maiwald A Maiwald G Marschner N Marx M von Minckwitz G Naß-Griegoleit I Possinger K Reiter A Sauerbrei W Schlake W Schmutzler R Schreer I Schulte H Schulz KD Souchon R Thomssen C Untch M Wagner U Weis J Zemmler T Interdisciplinary S3 guideline for diagnosis and therapy of breast cancer in women 2008 Berlin: German Cancer Society

[B13] Lancaster T Econometric methods for the duration of unemployment Econometrica 1979 47 939 956 10.2307/1914140

[B14] Schwentner L Wolters R Koretz K Wischnewsky MB Kreienberg R Rottscholl R Wöckel A Triple-negative breast cancer: the impact of guideline-adherent adjuvant treatment on survival—a retrospective multi-centre cohort study Breast Cancer Res Treat 2012 132 3 1073 1080 10.1007/s10549-011-1935-y 22205141

[B15] Joensuu H Gligorov J Adjuvant treatments for triple-negative breast cancers Ann Oncol 2012 23 6 vi50 10.1093/annonc/mds194 23012301

[B16] Aapro M Wildiers H Triple-negative breast cancer in the older population Ann Oncol 2012 23 6 vi52 vi55 23012304 10.1093/annonc/mds189

[B17] Hamaker ME Schreurs WH Uppelschoten JM Smorenburg CH Breast cancer in the elderly: retrospective study on diagnosis and treatment according to national guidelines Breast J 2009 15 26 33 10.1111/j.1524-4741.2008.00667.x 19141131

[B18] Wöckel A Kurzeder C Geyer V Novopasphenny I Wolters R Wischnewsky M Kreienberg R Varga D Effects of guideline adherence in primary breast cancer-A 5 year multi-center cohort study of 3976 patients Breast 2010 19 120 127 10.1016/j.breast.2009.12.006 20117932

[B19] Hebert-Croteau N Brisson J Latreille J Rivard M Abdelaziz N Martin G Compliance with consensus recommendations for systemic therapy is associated with improved survival of women with nodal negative breast cancer J Clin Oncol 2004 22 18 3685 3693 10.1200/JCO.2004.07.018 15289491

[B20] Hancke K Denkinger MD König J Kurzeder C Wöckel A Herr D Blettner M Kreienberg R Standard treatment of female patients with breast cancer decreases substantially for women aged 70 years and older: a German clinical cohort study Ann Oncol 2010 21 4 748 753 10.1093/annonc/mdp364 19825884

[B21] Schwentner L Wolters R Wischnewsky MB Kreienberg R Wöckel A Survival of patients with bilateral versus unilateral breast cancer and impact of guideline adherent adjuvant treatment: a multi-centre cohort study of 5292 patients Breast 2012 21 2 171 177 10.1016/j.breast.2011.09.007 21945313

[B22] Schwentner L Van Ewijk R Kurzeder C Hoffman I König J Kreienberg R Blettner M Wöckel A Participation in adjuvant clinical breast cancer trials: does study participation improve survival compared to guideline adherent adjuvant treatment? A retrospective multi-center cohort study of 9433 patients Eur J Cancer 2013 49 3 553 563 10.1016/j.ejca.2012.08.011 22959469

[B23] Varga D Wischnewsky M Atassi Z Wolters R Geyer V Strunz K Kreienberg R Wöckel A Does guideline-adherent therapy improve the outcome for early-onset breast cancer patients? Oncology 2010 78 3–4 189 195 20414007 10.1159/000313698

[B24] Janssen-Heijnen ML Maas HA Lemmens VE Houterman S Louwman WJ Verheij CD Coebergh JW The correlation of age and comorbidity with therapy and survival in cancer patients in North-Brabant and North-Limburg, 1995–2001 Ned Tijdschr Geneeskd 2005 149 30 1686 1690 16104115

[B25] DeMichele A Putt M Zhang Y Glick JH Norman S Older age predicts a decline in adjuvant chemotherapy recommendations for patients with breast carcinoma: evidence from a tertiary care cohort of chemotherapy-eligible patients Cancer 2003 97 9 2150 2159 10.1002/cncr.11338 12712466

[B26] Craft PS Buckingham JM Dahlstrom JE Beckmann KR Zhang Y Stuart-Harris R Jacob G Roder D Tait N Variation in the management of early breast cancer in rural and metropolitan centres: implications for the organisation of rural cancer services Breast 2010 19 5 396 401 10.1016/j.breast.2010.03.032 20452216

[B27] Goldhirsch A Wod WC Gelber RD Coates AS Thürliman B Senn HJ Progress and promise: highlights of the international expert consensus on the primary therapy of early breast cancer 2007 Ann Oncol 2007 18 7 1133 1144 10.1093/annonc/mdm271 17675394

